# Community perceptions of long-term mangrove cover changes and its drivers from a typhoon-prone province in the Philippines

**DOI:** 10.1007/s13280-021-01608-9

**Published:** 2021-08-23

**Authors:** Jay Mar D. Quevedo, Yuta Uchiyama, Ryo Kohsaka

**Affiliations:** 1 Graduate School of Environmental Studies, Tohoku University, Sendai, Japan; 2 Graduate School of Environmental Studies, Nagoya University, Nagoya, Japan

**Keywords:** Anthropogenic threats, Community perceptions, Local drivers, Mangrove cover change, Philippines

## Abstract

Mangrove forests are among the most productive ecosystems with important services such as food and livelihood provisions, recreations, and regulations (e.g., coastal protection) in local scales. At global scale, they are gaining salience for their carbon sequestration capacities, currently conceptualized as “blue carbon.” However, their essential benefits are reduced or lost when degraded. There is, therefore, a need to explore long-term mangrove cover change (MCC) and its underpinning drivers to develop sustainable management strategies. MCC has been analyzed extensively, including satellite images and field surveys, with drivers of changes frequently embedded in local contexts. Thus, in this study, MCC and the causal factors are evaluated at the local scale by gathering community perceptions in Eastern Samar, a typhoon-prone province in the Philippines, with a timeframe since the 1970s until the present. Results show that mangrove cover loss was observed following the occurrence of Typhoon Agnes in 1984 and Typhoon Haiyan in 2013 while conversion of mangrove areas to residential spaces was identified as a recurring driver of mangrove depletion from the early 1970s to 1990s. Study participants perceived that *natural threats* and *lack of law enforcement* were the leading proximate and underlying drivers of degradation, respectively. Respondents perceived that mangrove cover is increasing mainly due to successive reforestation programs coupled with stricter implementation of local ordinances in the sites. The results indicate the increased role of mangrove forests in disaster risk reduction and climate change mitigation strategies, while the perceptions of drivers change in long terms.

**Supplementary Information:**

The online version contains supplementary material available at 10.1007/s13280-021-01608-9.

## Introduction

Mangrove forests are among the most productive environments that provide diverse ecosystem services, namely provisioning (e.g., food source, livelihood options), regulating (e.g., coastal protection, coastal erosion), cultural (e.g., tourism, spiritual), and supporting (e.g., habitat, nutrient cycling) services (Primavera 2000; Menéndez et al. 2018; Friess et al. 2019; Spalding and Parrett 2019). In recent years, mangroves, along with seagrasses and tidal marshes, are increasingly referred to as “blue carbon” ecosystems, in recognition of their ability to sequester and store atmospheric carbon in the soil and living biomass (Nellemann et al. 2009). Such essential regulating service is gaining salience in the contexts of global climate change mitigation and adaptation agenda (Howard et al. 2017; Alongi 2020). Many international organizations such as the United Nations Environment Programme (UNEP), the International Union for Conservation of Nature (IUCN), and other international non-government organizations (NGOs) have provided policy recommendations, valuation methods, and financial support to improve the incorporation of blue carbon ecosystems in national climate change mitigation strategies and plans (UNEP 2014; Herr and Landis 2016; IUCN 2020).

To date, conservation and sustainable management of mangrove forests are being revisited and strengthened in the context of “blue carbon” science such as financial incentives to promote mangrove conservation for their blue carbon (e.g., Locatelli et al. 2014). Globally, there is also an increasing number of literatures that investigate MCC dynamics for the advancement of this research (e.g., Hermon et al. 2018; Sasmito et al. 2019; Friess et al. 2020). However, there is still so much needed to be done to improve existing understanding of the “blue carbon” concept by, for instance, identifying and addressing research gaps, modifying on-going strategies and programs, and amending present policies and frameworks (Friess et al. 2019; Macreadie et al. 2019).

Despite their local and global benefits, mangrove ecosystems are highly threatened, with anthropogenic threats (e.g., conversion to aquaculture ponds, urban developments, pollution, overexploitation) the leading proximate driver of deforestation (Richards and Friess 2016; Thomas et al. 2017). Previous studies on large-scale estimations of mangrove forests, including, for instance, historical rates of mangrove cover losses, and identification of threats have led to calls for improved mangrove conservation and protection practices in recent years (UNEP 2014; Hamilton and Casey 2016; Richards and Friess 2016; Thomas et al. 2017). The dynamics and changes in mangroves, frequently captured by mangrove cover change (MCC), are analyzed extensively, including satellite images and field surveys by scientists (e.g., Giri et al. 2011; Hamilton and Casey 2016; Bunting et al., 2018; Baloloy et al., 2020).

There are calls by the international scientific communities to capture the degradation and loss of biodiversity from direct drivers while reflecting indirect drivers (e.g., demographic, changes of lifestyles, values, and perceptions of resources) (IPBES 2018, 2019). Direct drivers of changes are frequently identified at the local scale contexts (of course, embedded in multi-dimension of global influences) (Kohsaka 2010). Thus, perceptions and behaviors of local communities are critical in addressing both direct and indirect drivers. The loss and degradation of mangrove forests will result in the reduction and/or loss of their valuable ecosystem services which in turn affect those local communities depending on them (Primavera 2000; IPBES 2019). There is, therefore, a need to understand community perspectives of MCC and its underpinning drivers to contextualize apt sustainable management strategies at the local scale with global implications.

In the Philippines, recent developments on mangroves that explicitly contributes to “blue carbon” research include, for example, carbon stock assessment and carbon sequestration potential (e.g., Dimalen and Rojo 2019; Salmo et al. 2019), payment, plans, and policy schemes (e.g., Thompson et al., 2014; Quevedo et al. 2021e; Song et al. 2021), and local stakeholders’ perceptions (e.g., Quevedo et al. 2020a; 2021a, b, d). Investigations of MCC dynamics are progressing as well, with many studies applying remote-sensing techniques and applications to decipher present and past mangrove losses (e.g., Hamilton and Casey 2016; Bunting et al. 2018; Buitre et al. 2019). Although these studies provide estimates of mangrove cover, its losses, and gains, drivers of such change are oftentimes not clearly distinguished. For instance, Long et al. (2014) have mapped the areal extent and spatial distribution of mangroves in the whole country from 1990 to 2010 using Landsat 30-m resolution data. They explained, to a certain extent, the potential MCC drivers (e.g., the occurrence of typhoons, aquaculture expansion) that influence the increase or decrease of mangrove coverage. Similarly, in the study conducted by Buitre et al. (2019), MCC was analyzed using time-series satellite imagery, and the authors discussed the possible impacts of tropical cyclones and human activities. However, their study showed a weak relationship between the drivers and vegetation change. Thus, their interpretations are limited to potential impacts and further recommended to examine other variables (e.g., the intensity of typhoons).

The advancement in remote-sensing applications allows rapid mapping and detection of MCC; however, identification of drivers is often not included and/or largely overlooked. This study aims to contribute to this gap by conducting perception surveys in coastal communities of Eastern Samar province, Philippines, where mangrove ecosystems are highly valued for their services (e.g., coastal protection, food provision), yet frequently exposed to anthropogenic and natural threats. Primarily, this work aims to (1) understand MCC dynamics in the study area, (2) determine the proximate and underlying drivers of MCC at the local level, and (3) show potential drivers of social perceptions of MCC, which are still limited information in the studied area. Evaluating MCC and identifying the drivers are important steps to understanding the current state of mangrove forests and to effectively develop or enhance conservation strategies that preserve and maximize their benefits. Such benefits include elements of blue carbon that are still mostly overlooked at the local scales (e.g., provincial, local), particularly at the grassroots level (Lukman et al. 2019; Quevedo et al. 2020a; Song et al. 2021). Moreover, this work further provides insights for ecosystem-based disaster risk reduction (Eco-DRR). This study can help stakeholders to protect and manage their mangrove forests to enable effective local strategies for disaster risk reduction and climate change mitigation, by deciphering the perceived MCC and its proximate (direct) and underlying (indirect) drivers.

## Theoretical framework

Human populations learn to distinguish how their activities and natural phenomena can affect them and their environments, allowing them to develop appropriate responses (Kohsaka and Rogel 2019). They have developed knowledge related to the places where they live since their land-use and management practices are known to affect or modify the landscape, which can help investigate landscape changes (Berkes et al. 2000; Bürgi et al. 2004; Aditya and Ganesh 2019). For instance, activities like charcoal production and urban expansions can negatively influence the state of the environment, whereas reforestation programs can positively affect the vegetation cover (Quevedo et al. 2021a). This knowledge allows them to build a perception of reality that is driven by socio-ecological, cultural, and economic values (Almeida et al. 2016) and direct experience and observation that are accumulated over time (Kohsaka and Rogel 2019). Therefore, in this context, the use of community perceptions to evaluate landscape changes and identify their drivers is possible (Gebrehiwot et al. 2010; Solomon et al. 2018). Additionally, research on local perceptions enables the collection of valuable information that supports policy makers in the development of conservation and sustainable management of local environments in different regional contexts (e.g., Martínez-Espinosa et al. 2020; Quevedo et al. 2020a; Kohsaka and Matsuoka 2015).

The use of community perceptions to evaluate changes in mangrove ecosystems and determine the causal factors has been applied by several studies, and, oftentimes, it is conducted alongside remote-sensing analysis. For instance, Gnanappazham and Selvam (2011) have monitored the distribution trends of mangrove forests in Pichavaran, South India using local perceptions and remote sensing. Their study showed that the increase of mangrove area as perceived by locals complements the data produced in the remote-sensing analysis. Contrastingly, the research conducted by Cornejo et al. (2005) in Navachiste-San Ignacio-Macapule lagoon complex, Sinaloa, Mexico has documented opposing results between the two approaches. They documented an increase in mangrove vegetation cover from remotely sensed data while local villagers had perceived it to be decreasing. Remote sensing may supply valuable information on MCC, but it might be a challenge in other areas where cloud cover is frequent or satellite images are unavailable due to other technical reasons. Thus, some studies rely on an entirely qualitative approach like the works of Nfotabong-Atheull et al. (2011), which assessed environmental changes in mangroves of Cameroon, Central Afrcia, and Owuor and Newton (2019), which determined the status and threats of mangroves of Mida Creek, Kenya.

Although MCC analysis using community perceptions has been applied and is well documented, the applications of the method remain a challenge. In general, since perceptions by locals are subjective processes, it can be based on their personal experiences and observation, level of comprehension, and manner of interpretation (Quevedo et al. 2021d). Their perceptions can be influenced by, for instance, demographic attributes (e.g., Owuor and Newton 2019; Quevedo et al. 2020a), knowledge and utilization (e.g., Puryono and Suryanti 2019; Quevedo et al. 2021b, c), and accessibility and proximity to resources (e.g., Uchiyama and Kohsaka 2016). Thus, collecting community perceptions to evaluate MCC or other land-use and land-cover change, in general, should be done carefully. Cornejo et al. (2005) stressed the importance of combining public perceptions with satellite imagery analysis to capture both quantitative and qualitative characteristics of MCC dynamics while Islam et al. (2018) collected several datasets to confirm their interview results. Following the same framework, the present study validated, compared, and supplemented the collected public’s perceptions with an array of secondary data such as remotely sensed data, climatological records, and other pertinent information.

## Materials and methods

This study collected two sets of data for MCC dynamics in Eastern Samar province, Philippines (section "Study area"). The primary data include community perceptions of MCC and the causal factors, while the secondary data include population density, archives of typhoon tracks, google earth images, remotely sensed data of mangrove cover, mangrove-related policies and management activities, and mangrove awareness and utilization. The former is validated, supplemented, and compared with, using the latter. The data collection procedures and analyses are presented in the following sections "Primary data collection and analysis" and "Secondary data collection and processing", respectively.

### Study area

The Philippines ranks 15th (world) and 6th (Asia) in terms of the most mangrove-rich countries based on the 2010 global mangrove forests distribution (Giri et al. 2011). The country holds at least half of the world’s approximately 65 mangrove species (Garcia et al. 2014). Earliest mangrove covers estimate that the country had as much as half a million hectares in 1918 and drastically reduced to 120,000 ha in 1994 which was mainly caused by anthropogenic activities such as local exploitation for fuelwood, conversion to aquaculture ponds, and unregulated developments (Primavera 2000). Natural disturbances such as storm surges caused by strong typhoons were documented to also contribute in the decrease of mangrove forest cover in the country (Garcia et al. 2014). Recent estimates of mangrove cover applying remotely sensed satellite observations showed an increase in 2010 with 256,185 ha (Long and Giri 2011) then reduced to 220,984 ha in 2016 (Bunting et al. 2018) and increased to 227,808 in 2019 (Baloloy et al. 2020). However, it is noted that there are discrepancies in the estimates from the different references since different satellite data and processing methods were used. For instance, Long and Giri (2011) used the Landsat data and Iterative Self-Organizing Data Analysis Techniques (ISODATA) clustering, whereas Bunting et al. (2018) utilized a combination of Synthetic Aperture Rada (SAR) from the Advanced Land Observing Satellite (ALOS) Phased-Array L-band Synthetic Aperture Radar (PALSAR) and optical satellite data from Landsat-5 Thematic Mapper (TM) and Landsat-7 Enhanced TM (ETM +). According to Baloloy et al. (2020), some of these misclassifications were observed in the Landsat-based estimates, where mangroves were classified as water within small mangrove stands.

**Fig. 1:**
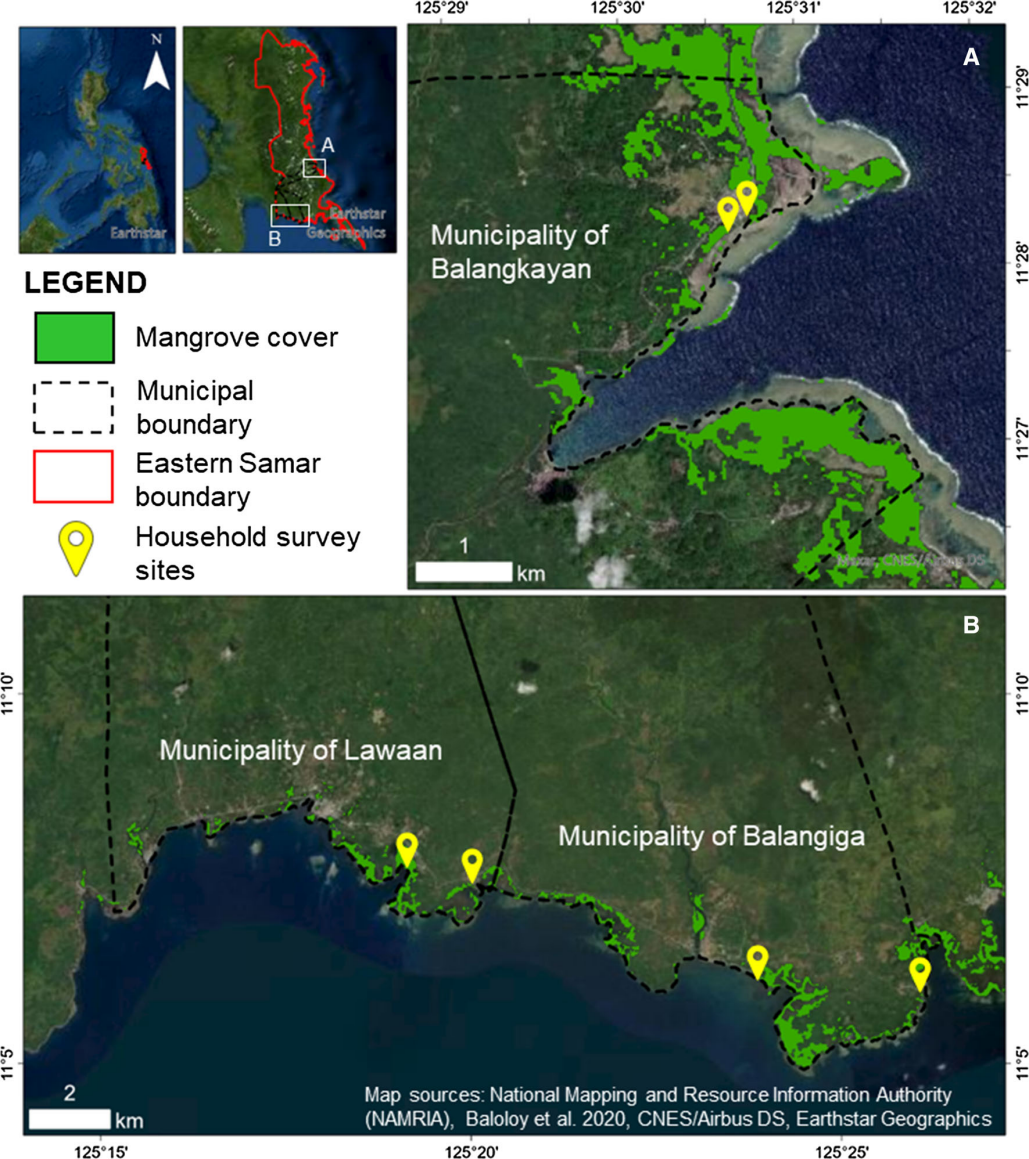
Location map of the study sites

This study explored the perceived MCC and its drivers by local communities in Eastern Samar province (Fig. 1). The province has a Type II climate based on the Modified Coronas Classification used by PAGASA (Philippine Atmospheric, Geophysical and Astronomical Services Administration), which is characterized by the absence of a dry season with a very pronounced maximum rain period from December to February, and a minimum precipitation during March to May. This province is on the Eastern coast of the country where tropical cyclones are known to make landfalls an average of 5.9 times per year between 1945 and 2013 (Takagi and Esteban 2015). Three municipalities, namely Lawaan, Balangiga, and Balangkayan were selected as study sites in the province (Fig. 1). These sites were chosen to complement the previous works of Quevedo et al. (2020a), where mangrove ecosystems are highly acknowledged by locals for their ecosystem services. Recent estimate of mangrove cover in the study areas is approximately 629.6 ha (Baloloy et al. 2020). The mangrove species include *Aegiceras, Avicennia*, *Bruguiera*, *Ceriops*, *Lumnitzera*, *Nypa*, *Rhizophora*, *Scyphiphora*, *Sonneratia*, and *Xylocarpus* (Alura and Alura 2016; Primavera et al. 2016).

### Primary data collection and analysis

#### Household surveys

The household surveys were carried out in three coastal villages of Lawaan, Balangiga, and Balangkayan in Eastern Samar province from November 18th to 20th, 2019. Field survey letter and permits stating the purpose and usage of data were sent to the respective local executive heads (municipal mayors) before the initiation of the surveys. The previous perception surveys conducted by Quevedo et al. (2020a) were used as a baseline for selecting the target areas with preference for older residents (Table 1). The respondents were selected randomly surveying one household in every five households where possible. Asking permission and stating the purpose of the survey were also carefully observed in this study. Field enumerators who were affiliated with the local government units assisted on conducting of the surveys.

**Table 1:** Socio-demographic characteristics of the respondents (*n* = 96)

Indicator	Frequency (No.)	Percentage (%)
Gender		
Male	46	48
Female	50	52
Age group		
< 30 years old	1	1
31–40	14	14
41–50	24	25
51–60	28	29
> 60 years old	28	29
Did not answer	1	1
Education		
No formal education	22	23
Primary	37	39
Secondary	31	32
Tertiary	5	5
Did not answer	1	1
Occupation		
Fisher	28	29
Farmer	5	5
Salaried individual	27	28
Housewife	24	25
Did not answer	12	13
Length of stay (*n* = 94)		
Below 5 years	1	1
6–10 years	11	11
11–15 years	7	7
16–20 years	12	13
Since birth	61	64
Did not answer	4	4

A total of 96 respondents were surveyed in this study. The survey sample size was carefully calculated at 95% confidence interval with a 10% sampling error using Cochran’s formula (Bartlett et al. 2001) adopted by Quevedo et al. (2020a, b; 2021b):

$$n = {{n_0} \mathord{\left/ {\vphantom {{n_0} {\left( {1 + {n_0}/N} \right)}}} \right. \kern-\nulldelimiterspace} {\left( {1 + {n_0}/N} \right)}},$$

where *n*_0_ = (*t*^2^ * *p* * *q*)/*d*^2^, and *t* = value of selected alpha level (in this case, *α* is 0.5, so the critical value is 1.96), *p* = estimated proportion of the population which has the attribute in question, *q* = 1 – *p*, and *d* = acceptable margin of error (in this study, 0.10), and *N* = populations size (36,952—total population size of the three surveyed municipalities). To equally cover the sites, the computed sample size (96) was divided into three (3), resulting in 32 respondents in each municipality. Out of the 96 respondents, 15 of them did not comment or share their perceptions of MCC.

#### Survey questionnaire

Semi-structured questionnaires were utilized as the primary method for data collection. The texts were translated to *Waray*, the local language of Eastern Samar, and composed of both open- and closed-ended questions (Online Appendix S1). The first section collected the name, age, gender, education, occupation, and residency (number of years living in the area) of the respondents (adopted from Quevedo et al. 2020a, b). The second section gathered information about participants’ perceptions of MCC. Respondents were asked to share the observed changes, drivers, and the year it happened (approximate timeline) and if possible, to locate these observed changes in the provided maps (see Online Appendix S1 for the maps used). The last section asked the respondents to rank a set of proximate and underlying drivers of MCC (derived from Munthali et al. 2019; Quevedo et al. 2020a) in order of importance (1 = most important to 5 = least important).

#### Data analysis

Descriptive statistics were used to describe the respondents’ socio-demographic characteristics and summarize their responses and ranking of drivers of MCC. The drivers perceived by respondents were ranked following the principle of weighted average using the ranking index adopted by Munthali et al. 2019:

$${\text{Index}} = \frac{{{R_n}{C_1} + {R_{n - 1}}{C_2} + \cdots {R_1}{C_n}}}{{\sum {{R_n}{C_1} + {R_{n - 1}}{C_2} + \cdots {R_1}{C_n}} }},$$

where *R*_*n*_ = value given for the least-ranked level (for example, if the least rank is the 8th, then *R*_*n*_ = 8, *R*_*n*−1_ = 7, *R*_1_ = 1); *C*_n_ = counts of the least-ranked level (in the above example, the count of the 8th rank = *C*_n_, and the count of the 1st rank = *C*_1_). Spearman’s rank (*ρ*) correlation and multiple regression analyses were also carried out. The former was used to examine two associations, perceived proximate and underlying drivers and perceived drivers and awareness level (retrieved from Quevedo et al. 2020a) while the latter was utilized to evaluate the influence of respondents’ socio-demographic attributes to perceived proximate and underlying drivers.

### Secondary data collection and processing

The perceived MCC and drivers were supplemented and validated using an array of secondary data from published studies, reports, online database, and personal communications. Historical images from Google Earth were collected to support, validate, and show the locations of perceived MCC. The retrieved images have the following acquisition dates: 07-28-2011 and 08-10-2019 for Lawaan, 08-31-2012 and 02-28-2019 for Balangiga, and 08-31-2012 and 08-26-2015 for Balangkayan. These images were selected based on their clarity and close proximity with the perceived time period of the respondents. These were later georeferenced by adding control points using the Georeference tools of ArcGIS Pro v2.6.2. Additionally, mangrove areal extent cover from 1960 to present were collected from various sources (Primavera 1995; Giri et al. 2011; Long et al. 2014; Hamilton and Casey, 2016; Bunting et al. 2018; Baloloy et al. 2020) to show the general trend across the Philippines and study sites.

Other datasets collected include previous records of population density (National Statistics Office 2013; Philippine Statistics Authority 2016) and typhoon tracks (Unisys Corporation and JTWC 2006; OCHA Philippines, 2018). Fisheries and environmental laws with relevance to mangroves were also collated from various sources (e.g., Department of Environment and Natural Resources 2013; Dieta and Arboleda 2004). Information on mangrove planting activities in the study sites was gathered from the municipal agriculture and tourism officers (pers. comm, 1 December 2020). Lastly, mangrove awareness of ecosystem services, retrieved from the same respondents from the study conducted by Quevedo et al. (2020a), was analyzed as a potential driver (along with socio-demographics) of locals’ perceptions.

## Results

The following sub-sections present the primary data collected in this study. Socio-demographic attributes of the respondents are shown in section “Socio-demographic attributes of the respondents” while the perceived MCC dynamics and its drivers are reflected in sections “Respondents’ perceived mangrove cover changes” and “Perceived drivers of mangrove cover change”, respectively.

### Socio-demographic attributes of the respondents

The overview of the socio-demographic characteristics of the respondents is presented in Table 1. The number of respondents in terms of gender is almost equal (52% female and 48% male) with an average age of 53 years old. The youngest respondent is 27 years old while the oldest is 86 years old. Respondents belonging to the “51 years old and above” age group account for 58% of the sample size (*n* = 96). The education profile shows that 39% of the respondents have attained primary school, 32% finished secondary level, and 5% reached tertiary level. In terms of occupation, 28% of the sample came from the ‘salaried individual’ group, which covers skilled workers, government employees, and part-time workers, while 29% are fishers, 25% are housewives, and 5% are farmers. More than half (64%) of the respondents have been residing in the studied area since birth.

### Respondents’ perceived mangrove cover changes

**Fig. 2:**
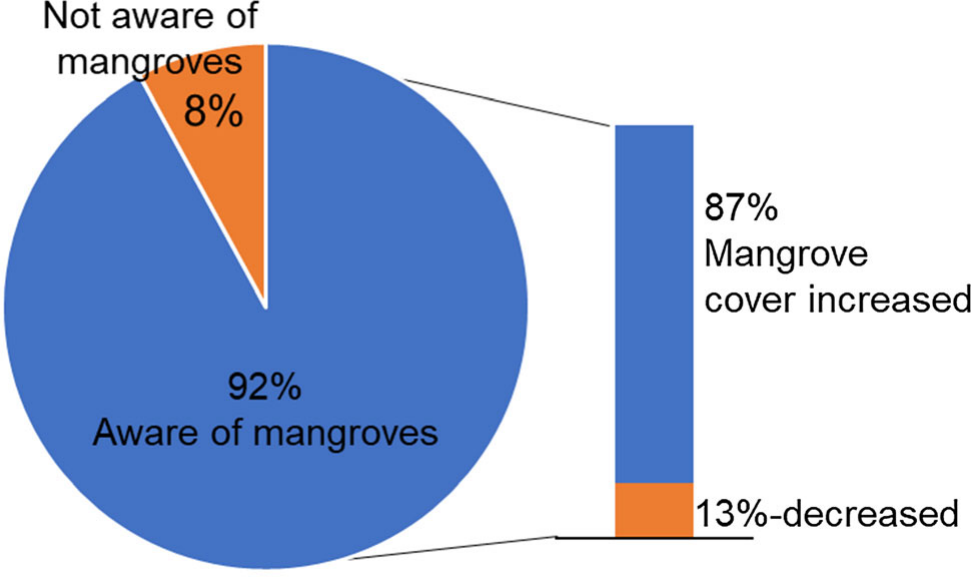
Mangrove awareness and perceived mangrove cover changed

**Fig. 3:**
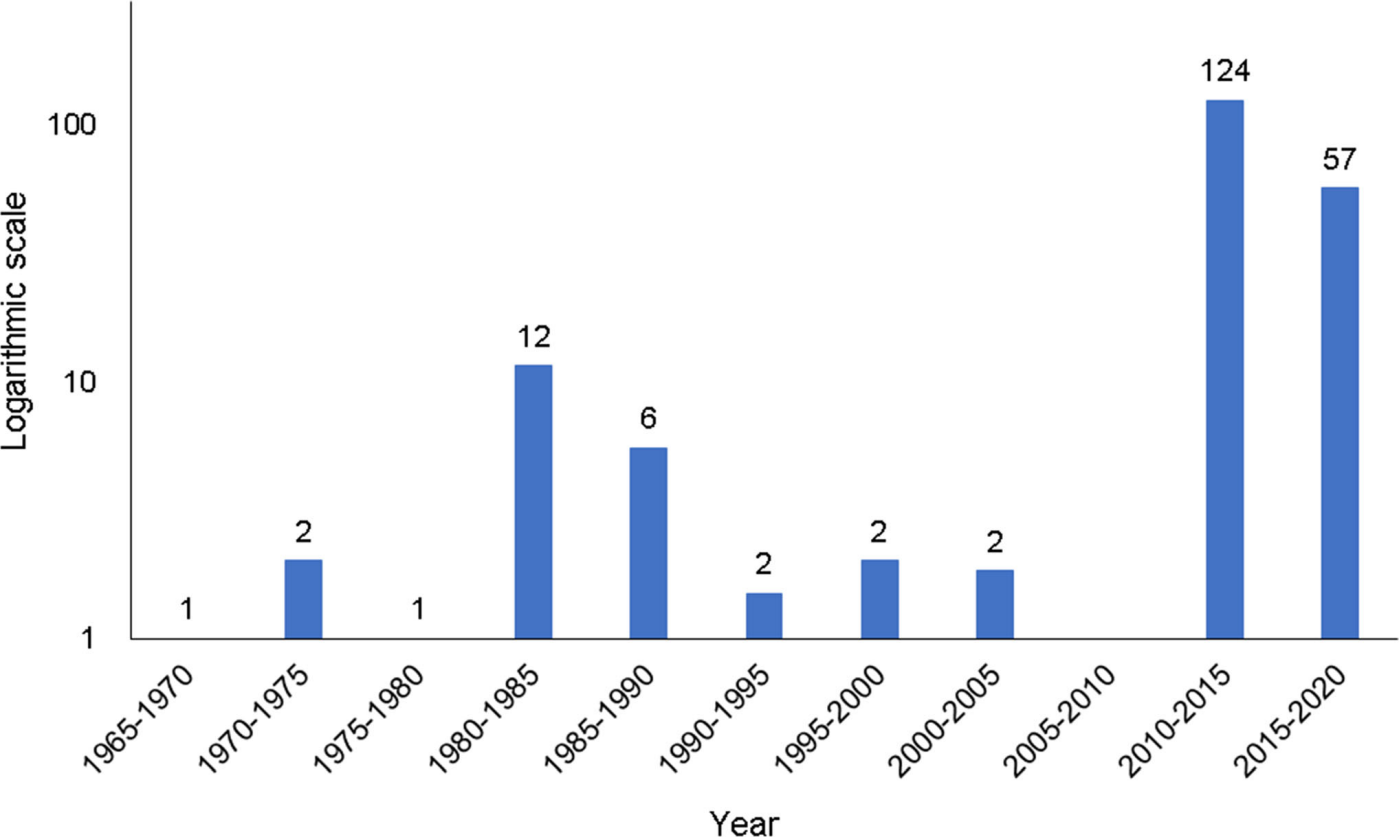
Frequency of perceived mangrove cover change using a logarithmic scale

Majority (92%) of the respondents were aware of the presence of mangrove forests in their municipalities, with 87% of them perceiving that mangrove cover has been recovering and increasing (Fig. 2). As further examination, this study collected community perceptions of MCC that are consolidated in Table 2. There were distinctive changes perceived over time. The general trend showed that there were significant MCC observed for several decades; however, impressions of recent MCC are stronger. Considering that past trends of MCC can be perceived less frequently, the logarithmic scale was used in Fig. 3, taking into account such characteristics of impression and perception of locals. Overall, mangrove areal extent cover was thick and abundant around the 1960s to 1970s, when settlements across the study sites were still few. From the 1970s to the 1990s, respondents perceived that the mangrove cover started to decline, with anthropogenic activities given as the main cause. For instance, in the early 1970s to 1980s, mangrove cutting for charcoal production was rampant in Lawaan while a portion of the coastal area in Balangiga with mangrove forests was bulldozed in 1981 in preparation for the port construction of a mining company. In Balangkayan, conversion to residential areas depleted the mangrove resources during the 1980s. From the 1990s to early 2000s, mangrove cover was perceived to be continuously declining across the study sites mainly due to the increasing number of settlements, which resulted in the conversion of mangrove areas to residential spaces and other land uses (e.g., fishponds in Lawaan) and increase in illegal activities (e.g., cutting of mangrove trees) coupled with lack of presence of local ordinances and weak law enforcement.

**Table 2:** Respondents’ perceptions of mangrove cover changes (*n* = 96)

Time period	Perceived mangrove cover change	Frequency (No.)	Percentage (%)	Age of the respondents
Balangiga				
1970s	Mangrove cover was abundant	1	3	60
1972	Fishing port was established	1	3	67
1981	Coastal area was bulldozed in preparation for the mining company	1	3	67
1989	Construction of port for the mining company	1	3	67
1997	Mining company closed	1	3	67
1990s	Construction of fishing port	1	3	36
2013	Destroyed due to super typhoon Haiyan	26	81	34–75
2014	Major rehabilitation works; mangroves recovering	24	75	34–75
2016	Fishing port was reconstructed	1	3	67
2017	Fishing port was reconstructed; residential areas moved further upland	1	3	36
2018	Mangrove cover is increasing	1	3	60
2019	Implementation of mangrove reforestation activities	23	72	34–75
	Did not answer/no comment	5	16	35–57
Lawaan				
Early 1970s–1980s	Mangrove cutting and charcoal production were rampant	1	3	55
1970–1990	Few settlements; abundant mangrove cover	1	3	83
Early 90s	Some mangrove areas were converted fishponds (4)	1	3	55
1995–2005	Illegal settlements increased	1	3	83
2006–2012	Mangrove cutting; LGU was not strict	1	3	83
2010	Fishponds were abandoned	1	3	55
2013	Natural regeneration of mangroves but destroyed when Haiyan hit the area	1	3	55
2013	Mangroves were destroyed due to super typhoon Haiyan	23	72	33–83
2014	Mangroves are recovering	10	31	33–83
2014–2016	Few settlements were built along the river	1	3	71
2017–2019	Mangroves are recovering, reforestation programs	18	56	46–86
	Did not answer/no comment	9	28	49–71
Balangkayan				
1967	Few settlements, thick mangrove canopy	1	3	72
1983	Mangrove areas were converted to residential areas	1	3	68
1984	Mangroves were washed out due to typhoon Agnes	10	31	44–86
1985	Seawall was constructed	4	13	44–68
2000	Few settlements, abundant mangrove cover	1	3	72
2001–2012	Increasing number of settlements; cutting of mangroves	1	3	72
2013	Mangroves were destroyed due to super typhoon Haiyan	28	88	31–86
2014–2019	Rehabilitation programs; mangroves are recovering	20	63	31–86
	Did not answer/no comment	3	9	27–43

**Fig. 4:**
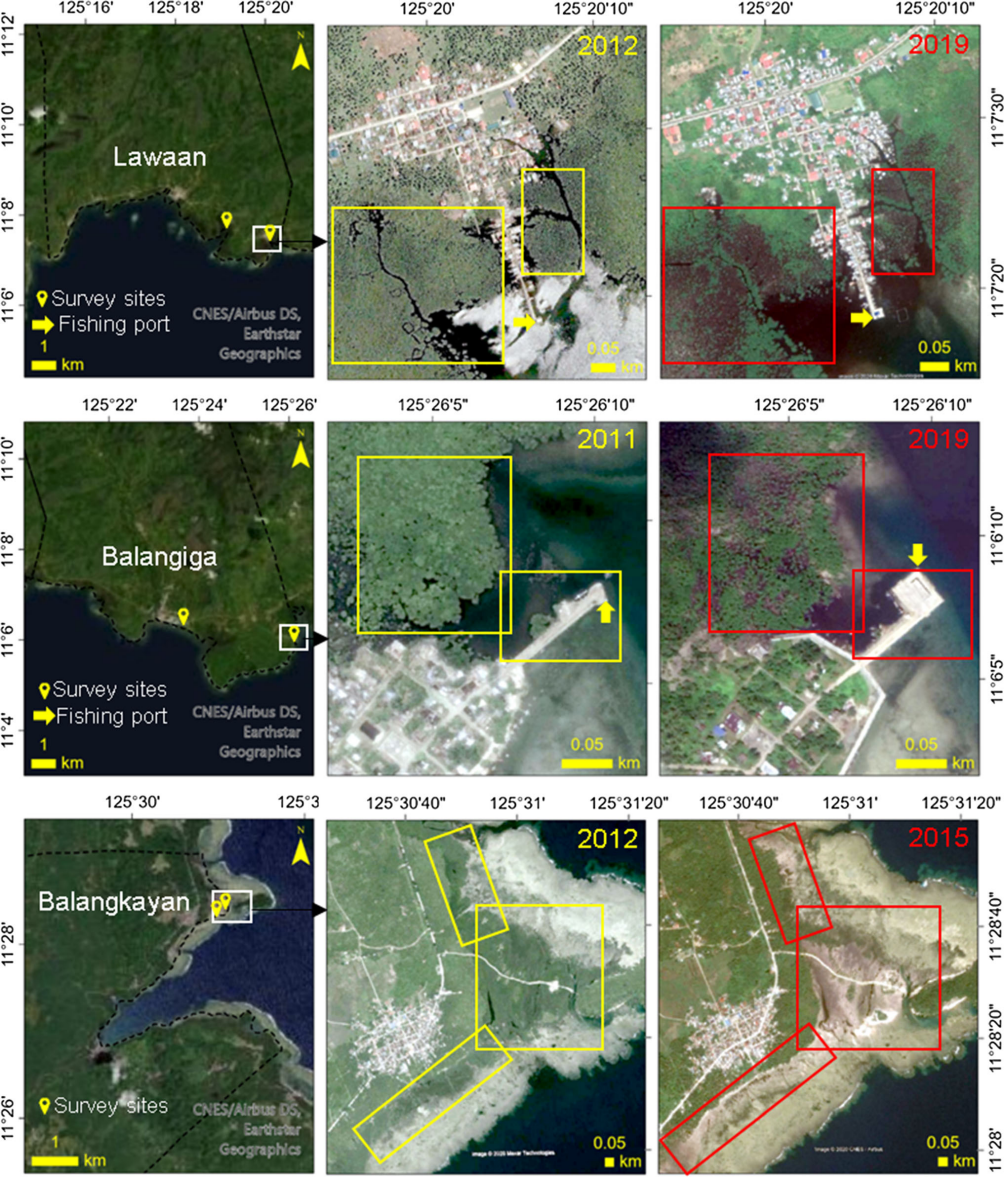
Mangrove cover of the study sites before and after Typhoon Haiyan

The impacts of natural threats to mangrove forest cover have also been perceived by the respondents. In Balagkayan, 31% of the respondents recollected the damages caused by Typhoon Agnes (Local Name—Undang) in 1984. They recalled how mangroves along the shores were destroyed, which led to the construction of a seawall in 1985 as alternative protection against strong waves and storm surges. Another documented event that caused massive wide-scale mangrove destruction across the study sites was Typhoon Haiyan (Local Name—Yolanda) in 2013. Eighty-one percent (81%), seventy-two percent (72%), and eighty-eight percent (88%) of the respondents in Balangiga, Lawaan, and Balangkayan, respectively, were able to remember and share how the typhoon washed away the mangrove areas along their coastlines. Figure 4 shows the sections within the study sites identified by the respondents that were damaged by the super typhoon.

Overall, respondents observed that the mangrove cover was recovering and increasing from 2014 to 2019. Seventy-five percent (75%), thirty-one percent (31%), and sixty-three percent (63%) of the respondents in Balangiga, Lawaan, and Balangkayan, respectively, attributed this recovery mainly to the implementation of major mangrove rehabilitation and reforestation programs. These projects were initiated by the local and national governments in response to the essential role of mangrove ecosystems in coastal protection against coastal hazards.

### Perceived drivers of mangrove cover change

This study also examined the drivers of MCC from the local community perspectives. The respondents identified and ranked 16 factors (8 proximate drivers and 8 underlying drivers) based on their importance as driving forces contributing to MCC in Eastern Samar (Table 3). The proximate drivers included “natural threats,” “coastal development,” “firewood,” “charcoal production,” “settlements,” “fishpond conversion,” “timber,” and “agriculture expansion.” The “natural threats” were ranked first as the most important driver followed by “coastal development” whereas the least important drivers were “fishpond conversion” (6th), “timber” (7th), and “agriculture expansion” (8th). With respect to the underlying drivers of MCC, the first (most) factor identified was the “lack of law enforcement,” while the least important (8th) was “political interferences.” Other underlying drivers perceived were “weak government policies,” “poverty,” “population growth,” “lack of financial resources,” “high cost of agriculture inputs,” and “urbanization” occupying the 3rd to 7th places, in that order.

**Table 3:** Perceived proximate and underlying drivers of mangrove cover changes (*n* = 96)

MCC proximate driver	No. of respondents per rank					Weight	Index	Rank
	1	2	3	4	5			
Natural threats	71	19	2	1	2	441	0.252	1
Coastal development	12	30	18	7	29	277	0.158	2
Firewood	14	12	9	9	52	215	0.123	3
Charcoal production	4	15	11	5	60	183	0.105	4
Settlements	2	5	23	18	47	182	0.104	5
Fishpond conversion	0	0	18	22	56	154	0.088	6
Timber	0	2	22	10	57	151	0.086	7
Agriculture expansion	0	0	9	37	47	148	0.085	8
MCC Underlying Driver								
Lack of law enforcement	14	22	27	8	25	280	0.162	1
Weak government policies	13	19	28	12	23	272	0.157	2
Poverty	21	15	1	3	56	230	0.133	3
Population growth	6	22	12	9	46	218	0.126	4
Lack of financial resources	7	18	10	20	41	218	0.126	4
High cost of agriculture inputs	1	7	15	28	43	177	0.102	6
Urbanization	3	7	9	31	40	172	0.099	7
Political interferences	2	4	13	22	54	163	0.094	8

*1* most important, *5* least important

Correlation analysis was carried out to examine the relationship between the perceived proximate and underlying drivers. Results show that the perceived level of importance of the proximate drivers can be attributed to respondent’s perceptions of the underlying drivers (Table 4). For instance, “coastal development,” which occupied the second place in the level of importance, was significantly correlated with “lack of law enforcement” (*ρ* = 0.280, *p* < 0.01) and “weak government policies” (*ρ* = 0.220, *p* < 0.01). This finding suggests that the respondents perceived that coastal development as a threat to mangrove ecosystems arises due to weak implementation and enforcement of policies. Similarly, significant correlations (*p* < 0.01) were recorded when participants’ perceptions of underlying drivers (“poverty,” “population growth,” and “lack of financial resources”) were attributed to “firewood,” “charcoal production,” and “settlements,” indicating the former (indirect drivers) may trigger the latter (direct drivers) to happen at the study sites. Interestingly, the most important proximate driver, “natural threats,” did not show any significant correlations, which suggests the reliability of this analysis since the former is of the natural domain and the underlying drivers in this study are all anthropogenic origin.

**Table 4:** Correlation analysis between perceived proximate and underlying drivers of MCC (*n* = 96)

Underlying driver (in order of most important)	Proximate driver (arranged from most [left] to least [right] important)							
	Natural threats	Coastal development	Firewood	Charcoal production	Settlements	Fishpond conversion	Timber	Agriculture expansion
1. Lack of law enforcement		0.280**						
2. Weak government policies		0.220*			-0.231*			
3. Poverty		0.262**	0.728**	0.739**	0.655**	0.545**	0.680**	0.310**
4. Population growth		0.284**	0.591**	0.607**	0.697**	0.445**	0.485**	0.254**
4. Lack of financial resources		0.299**	0.705**	0.671**	0.637**	0.460**	0.591**	0.354**
6. High cost of agriculture inputs			0.353**	0.374**	0.392**	0.413**	0.385**	0.249**
7. Urbanization			0.298**	0.449**	0.221*	0.304**	0.396**	
8. Political interferences			0.480**	0.538**	0.340**	0.397**	0.563**	0.290**

*,**Indicate significant correlations at *p* value < 0.05, *p* value < 0.01, respectively; only significant results are shown

Other factors that could potentially influence community perceptions of the proximate drivers of MCC were also explored in this study. Socio-demographic attributes (independent variables) of the respondents were regressed with their perceptions (dependent variables) of the drivers (Table S1). Results indicate that the former may affect locals’ recognition of the latter to some extent. For instance, demographic characteristics such as occupation influenced the perceived level of importance of five out of the eight direct drivers identified in this study—“coastal development” (*R*^*2*^ = 0.10, *p* < 0.01), “firewood” (*R*^*2*^ = 0.11, *p* < 0.01), “charcoal production” (*R*^*2*^ = 0.05, *p* < 0.09), “fishpond conversion” (*R*^*2*^ = 0.08, *p* < 0.03), and “agriculture expansion” (*R*^*2*^ = 0.07, *p* < 0.04). For the underlying drivers, only those that were closely related with socio-economic attributes could be regressed—“poverty” (*R*^*2*^ = 0.10, *p* < 0.01) and “population growth” (*R*^*2*^ = 0.06, *p* < 0.06). Another factor explored was the respondents’ awareness of mangrove ecosystem services. Using the data collected by Quevedo et al. (2020a), respondents’ perceptions of MCC proximate drivers were correlated with their awareness. Results show that there is a potential that participant’s observation of the direct drivers can be influenced by their knowledge of mangrove benefits (Table S2). Their awareness of the coastal protection services of mangrove forests, for instance, positively affects their recognition of “charcoal production” (*ρ* = 0.661, *p* < 0.01), “settlements” (*ρ* = 0.443, *p* < 0.10), and “fishpond conversion” (*ρ* = 0.666, *p* < 0.01) as proximate drivers of MCC.

## Discussions

The primary results (community perceptions) of this study are further elaborated and discussed by complementing, validating, and comparing with a suit of secondary data (e.g., remotely sensed data) to demonstrate how locals’ observations can be used to record MCC and its drivers over time. The following sections detail these discussions. Firstly section “Mangrove cover change dynamics”, temporal MCC is presented followed by section “Drivers of mangrove cover changes” the causal factors. The implications of this study are also discussed in the last section “Management and policy implications.”

### Mangrove cover change dynamics

**Fig. 5:**
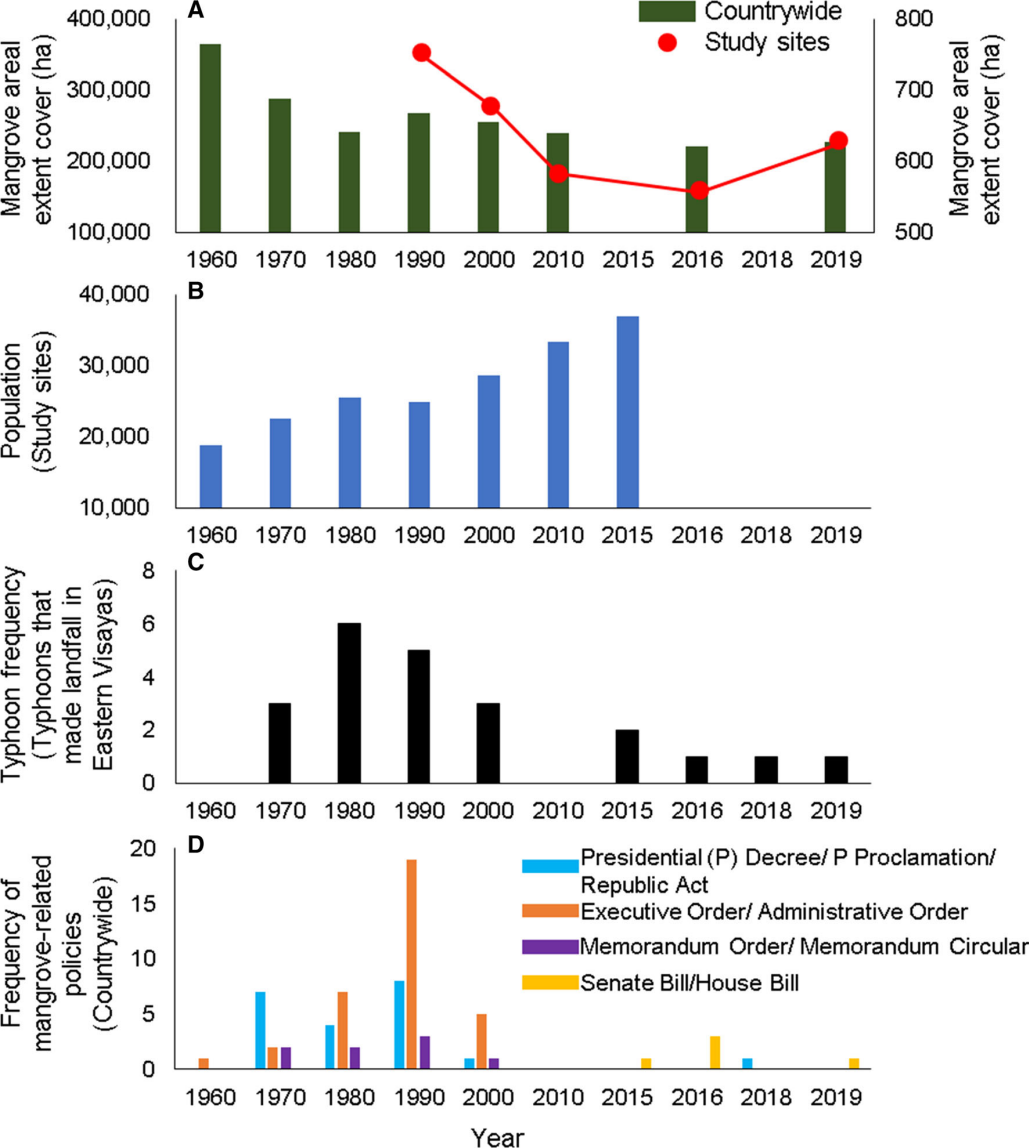
Secondary data used in this study: **A** Mangrove areal extent cover, **B** Population of the study sites, **C** Frequency of typhoons that made landfall in Eastern Visayas, and **D** Frequency of mangrove-related policies (implemented/planned). (Please see Methodologies section for data sources)

The results captured how mangrove forest cover in the study sites has been, in general, declining over time. The community perceptions collected are consistent with the observed mangrove areal extent cover based on the processed satellite images and remotely sensed data retrieved from Primavera (1995), Giri et al. (2011), Long et al. (2014), Hamilton and Casey (2016), Bunting et al. (2018), and Baloloy et al. (2020). The overall trend of mangrove cover in the study sites decreased from ~ 752 ha in 1990 to ~ 558 ha in 2016, before it gradually increased to ~ 630 ha in 2019 (Fig. 5A). The pattern observed in the study sites was reflective of the general mangrove cover trend observed for the whole country, suggesting that mangrove cover may have been declining as well during the earlier periods (1960 to 1980) (Fig. 5A). Mangrove cover in the country has decreased over time due to anthropogenic and natural threats, with the former being a more pronounced driver than the latter (Primavera 1995, 2000; Garcia et al. 2014). The same observations have been collected in this study, such that the perceived MCCs are governed by many causal factors, both natural and human-induced disturbances.

Considering the maximum age of the respondents (75 years old in Balangiga and 86 years old in Lawaan and Balangkayan), the earliest recorded period with perceived MCC is around the 1970s when mangrove areas were converted to residential spaces, reducing the thick and abundant cover observed during the 1960s (Table 2). This MCC driver has been observed and conducted towards the early 2000s, making it a recurring driver that contributes to mangrove depletion in the studied areas through time. These perceptions are validated and compared with historical population density (Fig. 5B) and mangrove cover (Fig. 5A). The population density shows an overall increasing trend from 1960 to 2015, while the mangrove cover reflects a decreasing trend from 1990 to 2016. The increasing population trend demands more residential spaces, which may have resulted in the clearing of mangrove areas along coastal zones. For example, in Balangkayan, one respondent shared how mangrove areas were cleared to cater to the increasing number of settlers from 2001 to 2012 (Table 3). The increasing number of settlers came with increasing demand for resource usage, resulting in overexploitation. In this study, respondents shared how mangroves were cut for charcoal production as an alternative income. This extractive activity was one of the major causes of the rapid depletion of mangroves in the country (Primavera 2000). In Busuanga Island, for instance, overexploitation was identified as one of the pressures stressing the mangrove ecosystems (Quevedo, Uchiyama, and Kohsaka 2021a).

In the Philippines, conversion of mangrove areas to aquaculture ponds was one of the leading causes of mangrove depletion from 1960 to 1990 when the fisheries sector was prioritized to boost the country’s economy (Primavera 2000; Dieta and Arboleda 2004). In Lawaan (this study), conversion to fishponds was recognized as well as a driver of mangrove depletion in the early 90s (Table 3). Coastal developments such as the construction of fishing ports in or along mangrove areas were identified as well (see Fig. 4), although respondents perceived this development as a necessity for easy access to and from the municipal waters where they fish (livelihood source).

From 2000 to 2016, mangrove cover in the study sites decreased from ~ 678 ha to ~ 558 ha (Fig. 5A). In the Balangiga-Lawaan mangrove stretch, the reduction rate was -40.22 ha per year from 2010 to 2016, which was mainly due to the super typhoon Haiyan in 2013 (Buitre et al. 2019). These quantitative values are consistent with the perceptions gathered in this study; respondents shared how the mangrove trees were severely damaged during this catastrophic event (Table 3). Figure 4 shows historical images retrieved from Google Earth of before (2011—Lawaan, 2012—Balangiga and Balangkayan) and after (2015—Balangkayan, 2019—Balangiga and Lawaan) super typhoon Haiyan hit the country. There is a noticeable amount of mangrove cover reduction in the areas identified by the respondents across the sites. About 34% of Balangiga-Lawaan mangrove area was damaged while 68% of the mangrove basal area in Balangkayan was destroyed (Alura et al. 2015).

The respondents perceived that the mangrove cover was recovering and increasing towards the present years, which complements the increasing trend observed in 2016 to 2019 from the remotely sensed data (Fig. 5A). This was mainly due, as perceived by the respondents, to the many mangrove rehabilitation and reforestation projects that were initiated and implemented in the study sites after 2013. These observations were accurate based on personal communications with the municipal agriculture and tourism officers (1 December 2020) of the study areas. In Balangiga, mangrove planting was conducted twice—2018 in Barangay (Brgy.) San Miguel and 2019 in Brgy. Bacjao. Mangrove rehabilitation was implemented thrice (2016, 2018, and 2020) in Lawaan and five times in Balangkayan during the periods 2014 to 2020. There was an increase in the frequency of mangrove reforestation programs in the study sites and, generally, in the Philippines because of recognition of the important role of mangrove trees in coastal protection against coastal hazards (Menéndez et al. 2018). This regulating ecosystem service has been recognized by many coastal communities especially in those areas affected by the super typhoon Haiyan in 2013 (Quevedo et al. 2020a, 2021b, d). This recognition of the importance of mangrove ecosystems in protection against coastal hazards (e.g., storm surges) paved the way for the increased presence of policies being filed (e.g., National Coastal Greenbelt Programs) at the national level (Fig. 5D).

### Drivers of mangrove cover changes

This study identified anthropogenic and natural drivers of MCC, similar to many studies conducted in the Philippines (e.g., Garcia et al. 2014; Long et al. 2014; Buitre et al. 2019) and globally (e.g., Giri et al. 2011; Hamilton and Casey 2016; Friess et al. 2019). Anthropogenic activities, as perceived in this study, greatly influenced the reduction of mangrove forests in the earlier periods (the 1960s to early 2000s). The impacts of direct anthropogenic drivers are influenced by the presence of indirect drivers (e.g., demographic, economic, socio-cultural drivers) (IPBES 2018). For instance, conversion to fishponds reduced the mangrove forest abundance when earlier policies in the country were directed towards the boosting of the fisheries sector (Primavera 2000). Meanwhile, illegal harvesting of mangrove trees for fuelwood and charcoal production and conversion to residential spaces proliferated due to poverty and lack of financial resources coupled with an increasing population. During the late 1990s, the number of unsanctioned activities (direct drivers) decreased when policies (indirect driver) that promote conservation and protection of mangroves increased (Fig. 5D) and with stricter implementation, resulting in mangrove expansion in the country (Dieta and Arboleda 2004). Although human-induced threats are less perceived in recent years (the late 2000s), their potential impacts on mangrove ecosystems are still highly acknowledged by the locals at the present time (Quevedo et al. 2020a).

In contrast, impacts of natural threats to mangrove forests tend to be perceived stronger for recent ones than the past events. The study sites, being geographically located in a typhoon-prone province in the Eastern side of the Philippines, are very vulnerable to tropical cyclones. The average frequency of typhoons making landfall in the Eastern Visayas is 2.2 per year (Fig. 5C) while the whole Eastern coast of the country is frequented by typhoons on average 5.9 times per year between 1945 and 2013 (Takagi and Esteban, 2015). However, despite the frequency of typhoon landfalls, the community was only able to identify two typhoons, Typhoon Agnes in 1984 and Typhoon Haiyan in 2013. Based on Japan Meteorological Agency (JMA) and Joint Typhoon Warning Center (JTWC), the former attained minimum central pressure of 925 hPa and 10-minue sustained winds of 105 kts (195 kph) while the latter attained a minimum central pressure of 910 hPa and a 10-min sustained winds of 110 kts (204 kph) (Kitamoto 2005; Paciente 2014; Villalba and Cruz 2017). Results of the surveys showed that the impact of 2013 typhoon was perceived more than the 1984 typhoon (Table 2). These findings suggests that (1) more respondents might have remembered vividly the more recent (2013) typhoon, including the damages, compared with the older one, given the relatively young age of the respondents (Table 2); (2) individual perceptions of the impacts of typhoons on mangrove areas may vary depending on its effect on their personal lives, as documented in the perception studies conducted by, for instance, Delfino et al. (2015) and Quevedo et al. (2020a); and/or (3) the intensity of typhoons is more pronounced towards recent years. The first and second points can be linked to an individual’s coping mechanisms, knowledge, and/or behavior towards typhoons and its impacts (Raphael 1986; Zhang et al. 2017; Wu et al. 2020). The respondents shared how Typhoon Haiyan damaged their properties and livelihoods. Typhoon Haiyan killed more than 6,000 people and damaged properties worth approximately 894 million USD while Typhoon Agnes killed more than 1,000 individuals and destroyed properties worth approximately 96 million USD (UN DHA 1984; Paciente 2014). In terms of mangrove damages, the respondents clearly observed the destruction brought by Typhoon Haiyan (Fig. 4). According to the assessment conducted by Alura et al. (2015), about 150 ha of Balangiga-Lawaan mangrove stretch was heavily destroyed while 39 ha of the mangrove basal area in Balangkayan was severely damaged. There is no available information on the extent of mangrove damage following the aftermath of Typhoon Agnes. Based on the simulations conducted by Villalba and Cruz (2017) and Soria et al. (2016), the associated storm surge of Typhoon Agnes was 2–3 m while Typhoon Haiyan has 5–7 m, respectively. The difference in the associated storm surge caused variations in the damages, which in turn could influence individual’s perceptions. The third point can be attributed to an increasing effect of climate change where frequency of severe weather patterns and intensified typhoons have become more prominent towards the present years (Buitre et al. 2019). The results of the study conducted by Chen et al. (2020) showed that peak intensities of tropical cyclones are expected to increase in the future as a result of surface warming, which enhances sea surface heat flux. The results presented here, however, are limited within the scope of this study and the authors noted that there are other dynamics that may play a role in perceptions. For instance, Israel and Briones (2014) analyzed the interactions between natural disasters and household income in the Philippines. Their study showed that an individual’s economic status (e.g., poor households) influences their coping strategies. Another example is from the case study conducted by Yao et al. (2021) in Ningbo, China, where they documented that environmental values and government’s disaster management ability significantly influenced respondent’s risk perceptions of typhoons.

### Management and policy implications

Mangrove ecosystems in the study sites are an integral source of food and livelihood and serve as natural protectors against coastal hazards brought by, for instance, strong typhoons (Primavera et al. 2016). When these coastal resources are degraded or damaged, their valuable ecosystem services are reduced or lost in the process (Primavera 2000). Thus, this study gathered community perceptions of MCC and its drivers, which are critical information towards achieving sustainable mangrove management. Having said this, there is a need to conserve these public perceptions of MCC particularly at the local level, where sharing and transferring knowledge among different stakeholders is constrained by institutional and technical capacities and capabilities (Quevedo, Uchiyama, and Kohsaka 2021a).

The MCC caused by anthropogenic threats can be, in theory, reduced and halted if corresponding policies and programs are effective and enforced efficiently. In this study, MCC were triggered by anthropogenic activities, which in turn were influenced by the underlying drivers. Lack of law enforcement and weak government policies are the top two indirect drivers that encourage the proliferation of unsanctioned activities (e.g., illegal cutting, firewood, and charcoal production). Despite the existing plans (e.g., Coastal Resource Management Plans) and marine protected areas in place, the implementation is weak and local ordinance enforcement is lacking (Buitre et al. 2019; Quevedo et al. 2021e). There is, therefore, a need for LGUs to strengthen and/or enhance existing plans and ordinances to continue the conservation and protection of mangrove forests. Strategies such as information and educational campaigns can be developed to reduce the gap between an individual’s knowledge and behavior towards mangroves. Primavera et al. 2012 documented that increasing communities’ awareness of mangrove benefits promotes the protection and conservation of these ecosystems. Additionally, Quevedo et al. (2020a) were able to document that public’s perceptions of general threats to mangroves can be influenced by the presence of local ordinances.

The resilience of mangroves to natural threats can be influenced by their overall ecological health and condition. For instance, Alongi (2008) documented that mangroves have a variety of key features (e.g., large reservoir of below-ground nutrients and complex and highly efficient biotic controls) that contribute to their resilience against disturbances. However, if they are heavily damaged (e.g., anthropogenic activities), these features will require time to recover. In a similar vein, this study was able to document evidence of reduced mangrove resilience; the natural regeneration of mangroves, which was observed in the abandoned fishponds, was damaged during the 2013 catastrophic event (see Table 2). The impact of the typhoon was too large and intense for the regenerated mangroves to withstand. The effects of natural threats to mangrove forests can also be reduced, for instance, by following a science-based approach in mangrove reforestation activities. In the study conducted by Primavera et al. (2016), mangrove plantations in Eastern Samar coastal municipalities were mostly *Rhizophora* species in the seaward sections, which have a low resistance to strong winds and waves (e.g., Carlos et al. 2015). Hence, it was severely damaged by super typhoon Haiyan. The investigation of Villamayor et al. (2016) in Eastern Samar also generated similar findings; *Rhizophora* plantations were heavily destroyed by Typhoon Haiyan. The results of these investigations corroborate with the perceptions gathered in this study. Thus, to reduce mortality rates of the mangroves being reforested, science-based mangrove rehabilitation should be adapted to ensure that proper mangrove species are planted in the correct zones. The works of Primavera et al. (2012) and Camacho et al. (2020), for instance, have highlighted the importance of science-based approach in the success of mangrove rehabilitation and reforestation programs.

## Conclusions

To address the international calls to identify both direct and indirect drivers, the perceptions of local communities related to MCC are captured and analyzed in this study. Evaluating MCC and identifying the drivers are important steps in understanding the current state of mangrove forests, which in turn support the policy makers to effectively develop or enhance conservation and management strategies at the local level. This study analyzed MCC dynamics in three municipalities of a typhoon-prone province in the Philippines from locals’ perspectives. Long-term changes, from the 1960s to 2019, were documented based on community perceptions. The proliferation of proximate (direct) drivers (e.g., firewood, charcoal production) identified in this study during the earlier periods (the 1960s to early 2000s) is greatly associated with the presence of underlying (indirect) drivers such as lack of law enforcement and weak government policies. Although the occurrence of anthropogenic threats is less observed in the recent periods (the late 2000s), they are still highly acknowledged by the locals at the present time (Quevedo et al. 2020a).

Drivers of such changes have evolved over time, with anthropogenic threats as more pronounced drivers in the earlier periods, while impacts of natural threats are observed more frequently in recent years. The results of this study follow the general trend observed in the overall country. For instance, human-induced disturbances such as conversion of mangrove areas to aquaculture ponds were prolific in the earlier periods, when fishing industry was the priority of the country (Primavera 2000) while occurrence of more intensified typhoons is more common in the more recent years (Mei and Xie 2016). Based on the cluster analysis conducted by Mei and Xie (2016), typhoons in the northwest Pacific have intensified by 12%–15%, with the proportion of storms under categories 4 and 5 doubled or even tripled. They associated the increased intensity of landfalling typhoons with strengthened intensification rates, which in turn caused by enhanced sea surface warming. The occurrence of these more intensified natural calamities, as induced by climate change, can contribute to the decline of mangrove plantations (Ward et al. 2016). Mangrove protection, conservation, rehabilitation, and reforestation programs in the country have progressed in the recent years, which contributed to recovery of mangrove strands (e.g., Primavera et al. 2012). This was also documented in this study particularly from 2014 onwards (see Table 2), when mangroves gained renewed national interest following the devastation of Typhoon Haiyan in 2013. Thus, there is an opportunity for the LGUs at the study sites to solidify their coastal management plans in terms of conservation measures and sustainable management of these ecosystems following the national-level programs or mandates.

Finally, there are certain methodological implications that the primary data presented here complement the secondary data used, which corroborates the findings of other scientific literatures cited in this work. This is a vital confirmation for other researchers who are considering the use of community perceptions to analyze MCC when GIS-related approaches are limited or not feasible. Moreover, this work highlighted that community perceptions can record MCC and identify the causal factors, which are essential information to gather in formulating threat-specific management strategies of mangrove ecosystems.

## Supplementary Information

Below is the link to the electronic supplementary material.

Supplementary file1 (PDF 2499 kb)
