# Correction: Community perceptions of long-term mangrove cover changes and its drivers from a typhoon-prone province in the Philippines

**DOI:** 10.1007/s13280-023-01915-3

**Published:** 2023-09-13

**Authors:** Jay Mar D. Quevedo, Yuta Uchiyama, Ryo Kohsaka

**Affiliations:** 1 Graduate School of Environmental Studies, Tohoku University, Sendai, Japan; 2 Graduate School of Environmental Studies, Nagoya University, Nagoya , Japan

**Correction to: Ambio (2022) 51:972–989** 10.1007/s13280-021-01608-9

The article “Community perceptions of long-term mangrove cover changes and its drivers from a typhoon-prone province in the Philippines”, written by Jay Mar D. Quevedo, Yuta Uchiyama and Ryo Kohsaka, was originally published Online First without Open Access. After publication in volume 51, issue 4, page 972–989, the author decided to opt for Open Choice and to make the article an Open Access publication. Therefore, the copyright of the article has been changed to © The Author(s) 2021 and the article is forthwith distributed under the terms of the Creative Commons Attribution 4.0 International License, which permits use, sharing, adaptation, distribution and reproduction in any medium or format, as long as you give appropriate credit to the original author(s) and the source, provide a link to the Creative Commons licence, and indicate if changes were made. The images or other third party material in this article are included in the article's Creative Commons licence, unless indicated otherwise in a credit line to the material. If material is not included in the article's Creative Commons licence and your intended use is not permitted by statutory regulation or exceeds the permitted use, you will need to obtain permission directly from the copyright holder. To view a copy of this licence, visit http://creativecommons.org/licenses/by/4.0/.

The original article has been corrected.

