# Supplementary material for: Community perceptions of long-term mangrove cover changes and its drivers from a typhoon-prone province in the Philippines
Source: Ambio. 2021 Aug 23;51(4):972–89. doi: 10.1007/s13280-021-01608-9 (PMC8847476; doi:10.1007/s13280-021-01608-9)
Supplement: Supplementary file 1 — Supplementary file1 (PDF 2499 kb) [file 13280_2021_1608_MOESM1_ESM.pdf]

Ambio

Supplementary Information
This supplementary information has not been peer reviewed.

Title: **Community perceptions of long-term mangrove cover changes and its drivers from a typhoon-prone province in the Philippines**

Authors: Jay Mar D. Quevedo, Yuta Uchiyama, Ryo Kohsaka

Appendix S1. Survey Questionnaire.

Name:______________________________ Age:____
Sex: [ ]M [ ]F
Address:____________________________ Education:________________
Occupation:_________________ Length of stay in the community:[ ] Since birth [ ] below 5 yrs [ ]6-10 yrs [ ]11-15 yrs [ ] 16-20 yrs [ ] above 20 yrs

Guide questions for the land-use and land-cover changes using the map. Refer to next pages.

1. a. Do you know of any mangrove forests in your area? [ ] Yes [ ] No
   b .What has happened to mangrove forest cover in your community over the past few years?
   [ ] Increased [ ] Declined [ ] No change

2. Please **identify and mark the area(s) on the map** where you have **observed mangrove cover changes (MCC).**
Comments:____

3. When did it happen? (Please provide the **approximate timeline**) Were there any **laws, policies or ordinances** when it happened?

4. What was/were the land-use and land-cover (LULC) of the area **before the changes**? What is the **present LULC** of the area?

Proximate and underlying causes (drivers) of MCC (Modified from Munthali et al., 2019)

5. What do you think are the causes of LULC changes in your area? Please rank on a scale of 1 to 5; 5 = least important and 1 = most important.

**Proximate cause**	**Rank**					**Underlying driver**	**Rank**				
	**1**	**2**	**3**	**4**	**5**		**1**	**2**	**3**	**4**	**5**
Firewood						Poverty					
Charcoal production						Population growth					
Timber						Lack of financial resources					
Fishpond conversion						Lack of law enforcement					
Agriculture expansion						Weak government policies					
Coastal development						Urbanization					
Settlements						High cost of agriculture inputs					
Natural threats (i.e. typhoon)						Political interferences					
Other (please specify)						Other (please specify)					

Observed mangrove cover changes in your municipality.

Transitional change (prior and present): please tell us the most remarkable transitional change in the target sites and indicate the location where the change(s) happened.

YEAR: → YEAR: → YEAR: → YEAR:

↓

COMMENTS:____

____

____

YEAR: ← YEAR: ← YEAR: ← YEAR:

MUNICIPALITY OF BALANGKAYAN

100 by 100 m Map 8/10/2019

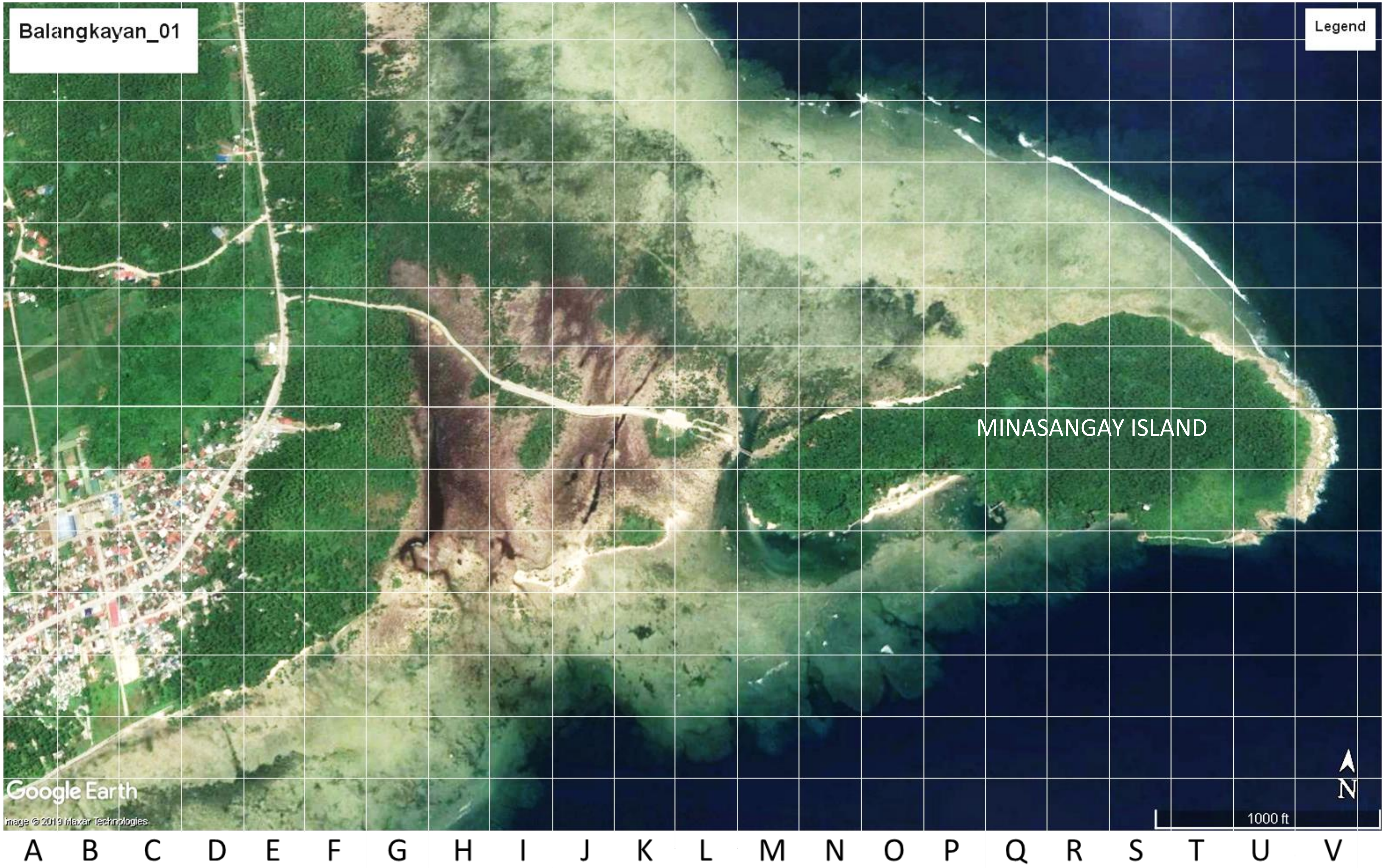

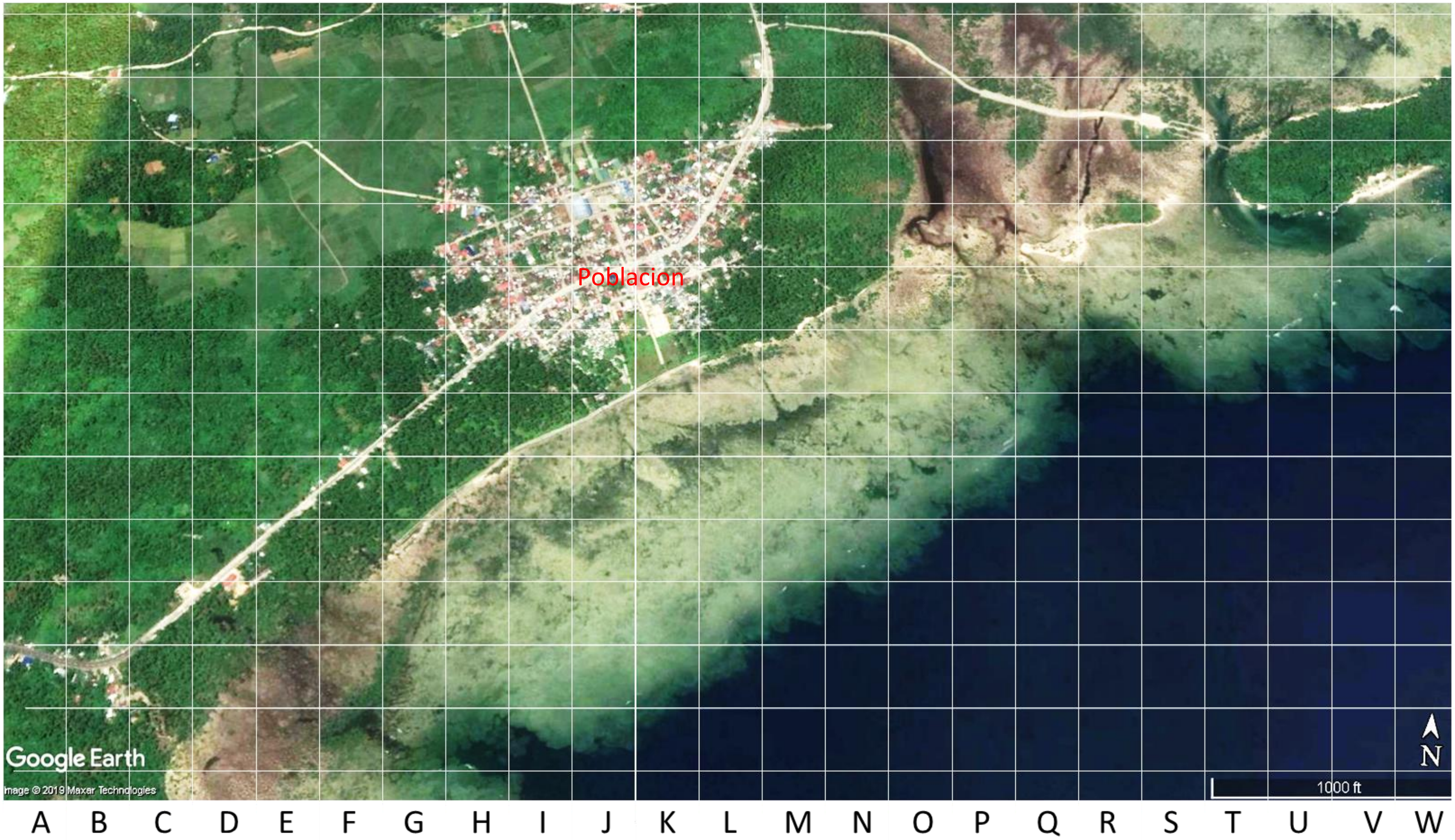
Poblacion
Google Earth
Image © 2019 Maxar Technologies
1000 ft
N
A B C D E F G H I J K L M N O P Q R S T U V W

MUNICIPALITY OF BALANGKAYAN

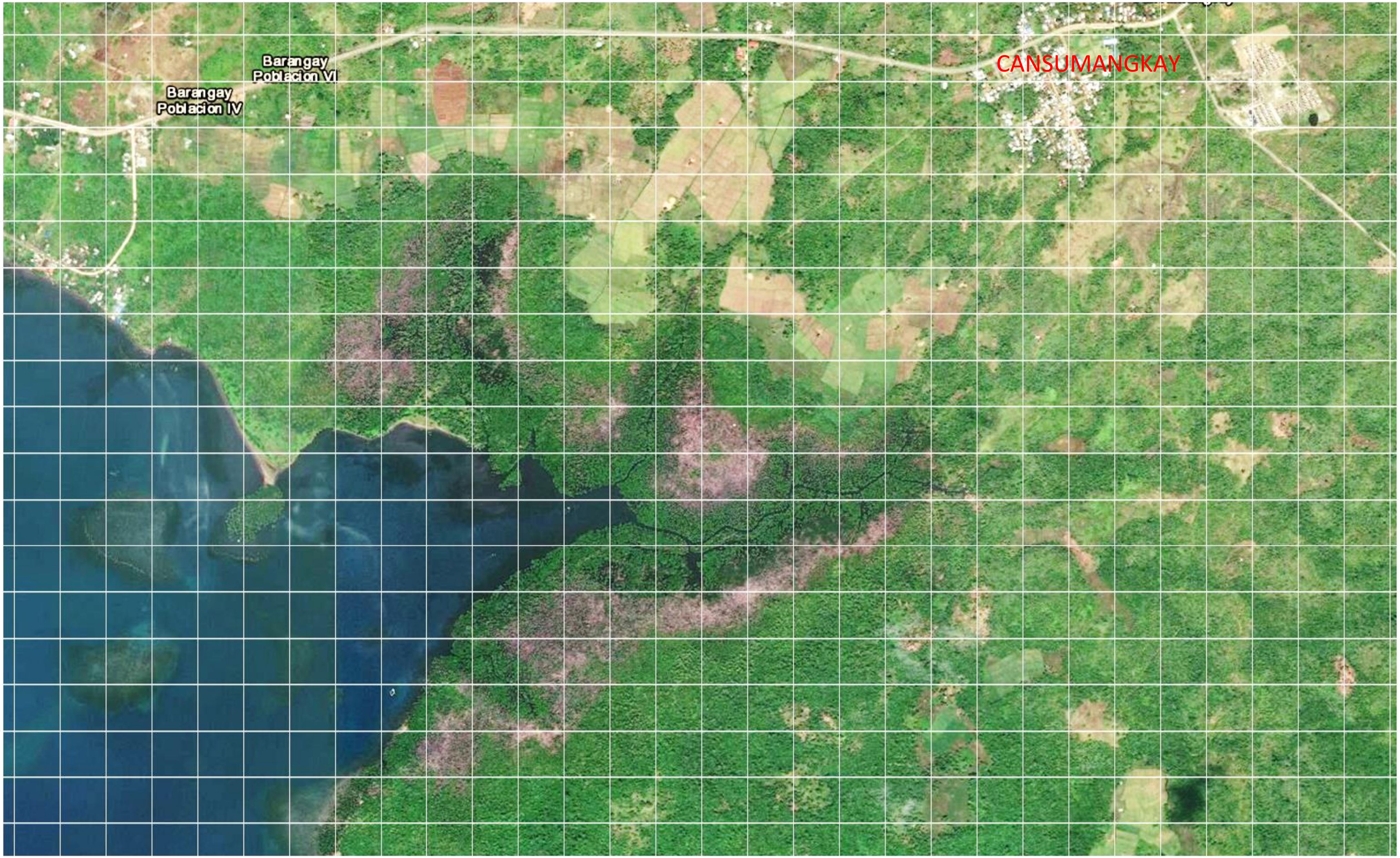

A B C D E F G H I J K L M N O P Q R S T U V W X Y Z AA AB AC AD

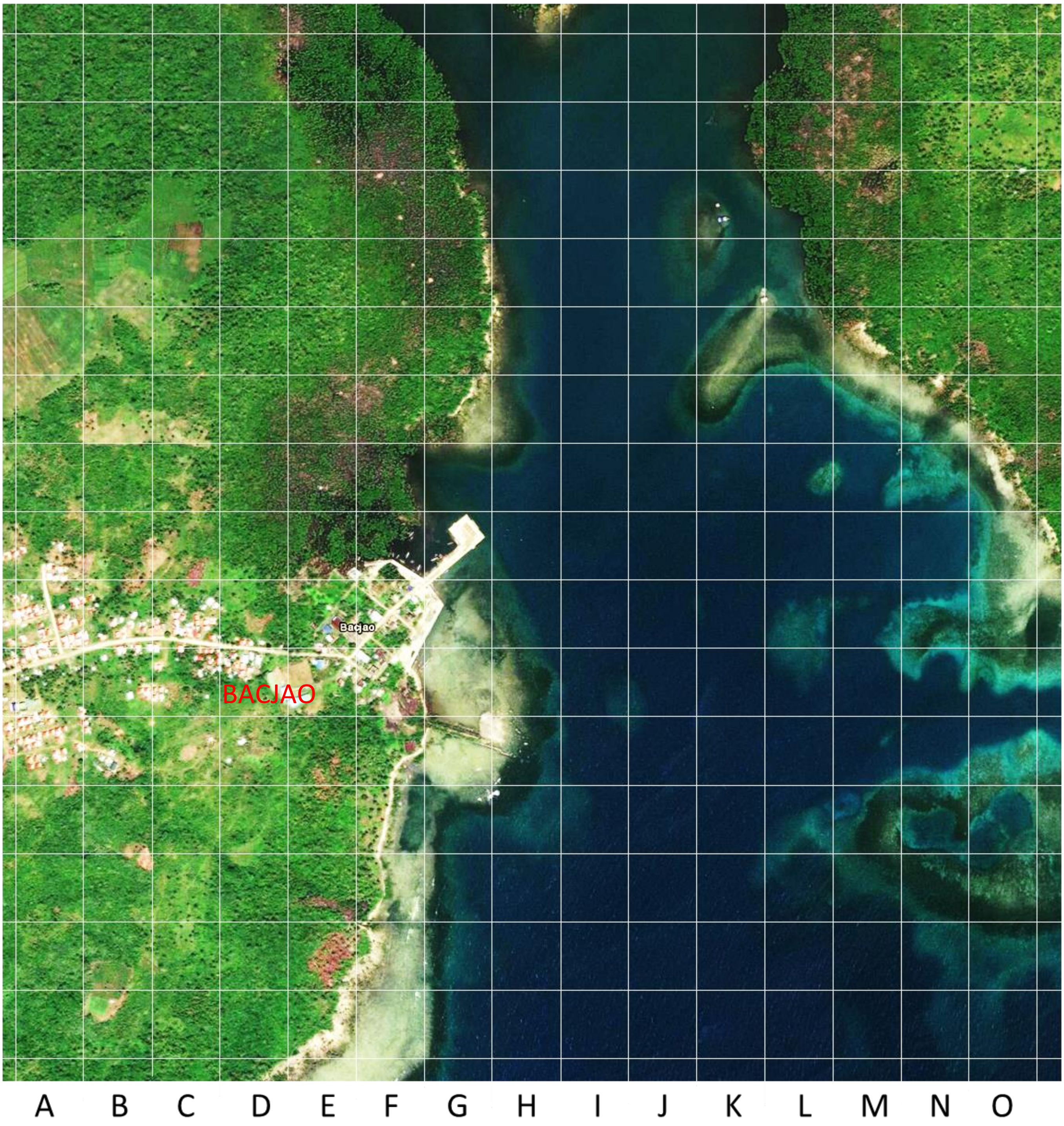

Bacjao
BACJAO
A
B
C
D
E
F
G
H
I
J
K
L
M
N
O

MUNICIPALITY OF LAWAAN

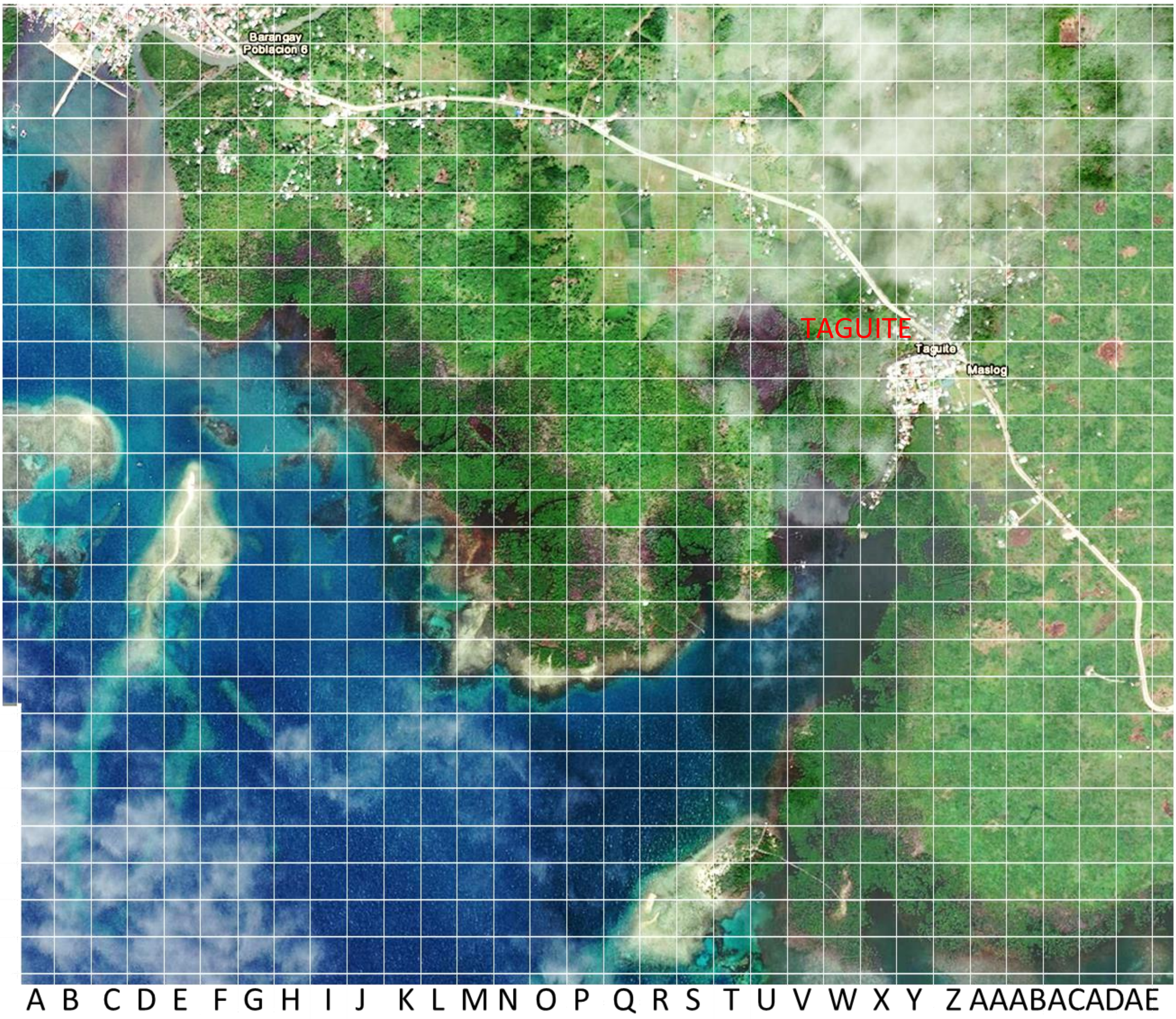

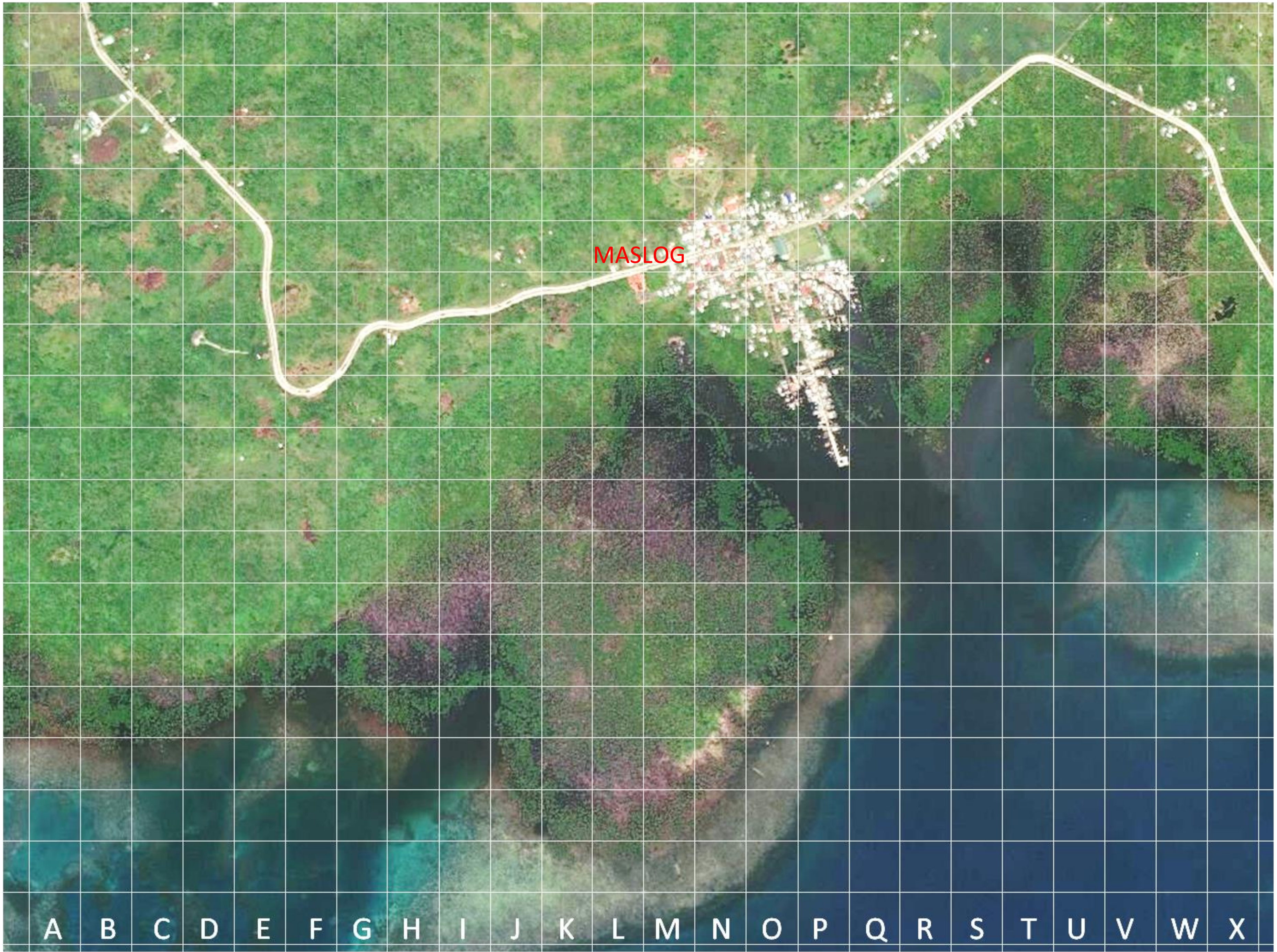
MASLOG
A B C D E F G H I J K L M N O P Q R S T U V W X

Table S1. Multiple regression analysis between respondents' socio-demographic attributes (independent variables) and perceived proximate and underlying drivers (dependent variables) (n = 96).

Regression Statistics	Level of importance of MCC proximate drivers (arranged from most [left] to least [right] important)							
	Natural threats	Coastal development	Firewood	Charcoal production	Settlements	Fishpond conversion	Timber	Agriculture expansion
Multiple R	0.25	0.43	0.44	0.37	0.36	0.41	0.34	0.40
Adjusted R Square	-0.04	0.10	0.11	0.05	0.04	0.08	0.03	0.07
p-value	0.68	**0.01**	**0.01**	**0.09**	0.13	**0.03**	0.18	**0.04**
	Level of importance of MCC underlying drivers (arranged from most [left] to least [right] important)							
Regression Statistics	Lack of law enforcement	Weak government policies	Poverty	Population growth	Lack of financial resources	High cost of agriculture inputs	Urbanization	Political interferences
Multiple R	0.34	0.19	0.43	0.38	0.36	0.29	0.36	0.32
Adjusted R Square	0.02	-0.06	0.10	0.06	0.04	-0.01	0.04	0.01
p-value	0.20	0.93	**0.01**	**0.06**	0.13	0.43	0.13	0.27

Note: values in bold indicates significant regressions at p-value < 0.10.

Table S2. Correlation analysis between respondents' perceived proximate drivers and their awareness of mangrove ecosystem services (n = 16).

Mangrove ecosystem services	Proximate driver (in order of most important)							
	Natural threats	Coastal development	Firewood	Charcoal production	Settlements	Fishpond conversion	Timber	Agriculture expansion
Food source								
Coastal protection			0.502*	0.661***	0.443*	0.666***	0.457*	0.545**
Air purification						0.655***		
Water purification						0.678***		
Carbon sequestration							0.496*	
Recreational site						0.489*		

*,**,*** indicates significant correlations at p-value < 0.10, p-value < 0.05, and p-value < 0.01, respectively; only significant results are shown.
